# Lipid Alterations in Glioma: A Systematic Review

**DOI:** 10.3390/metabo12121280

**Published:** 2022-12-16

**Authors:** Khairunnisa Abdul Rashid, Kamariah Ibrahim, Jeannie Hsiu Ding Wong, Norlisah Mohd Ramli

**Affiliations:** 1 Department of Biomedical Imaging, Faculty of Medicine, Universiti Malaya, Kuala Lumpur 50603, Malaysia; 2 Department of Biomedical Science, Faculty of Medicine, Universiti Malaya, Kuala Lumpur 50603, Malaysia

**Keywords:** lipids, glioma, lipid biomarker, bioactive lipid compounds, lipid signals

## Abstract

Gliomas are highly lethal tumours characterised by heterogeneous molecular features, producing various metabolic phenotypes leading to therapeutic resistance. Lipid metabolism reprogramming is predominant and has contributed to the metabolic plasticity in glioma. This systematic review aims to discover lipids alteration and their biological roles in glioma and the identification of potential lipids biomarker. This systematic review was conducted using the preferred reporting items for systematic reviews and meta-analyses (PRISMA) guidelines. Extensive research articles search for the last 10 years, from 2011 to 2021, were conducted using four electronic databases, including PubMed, Web of Science, CINAHL and ScienceDirect. A total of 158 research articles were included in this study. All studies reported significant lipid alteration between glioma and control groups, impacting glioma cell growth, proliferation, drug resistance, patients’ survival and metastasis. Different lipids demonstrated different biological roles, either beneficial or detrimental effects on glioma. Notably, prostaglandin (PGE2), triacylglycerol (TG), phosphatidylcholine (PC), and sphingosine-1-phosphate play significant roles in glioma development. Conversely, the most prominent anti-carcinogenic lipids include docosahexaenoic acid (DHA), eicosapentaenoic acid (EPA), and vitamin D3 have been reported to have detrimental effects on glioma cells. Furthermore, high lipid signals were detected at 0.9 and 1.3 ppm in high-grade glioma relative to low-grade glioma. This evidence shows that lipid metabolisms were significantly dysregulated in glioma. Concurrent with this knowledge, the discovery of specific lipid classes altered in glioma will accelerate the development of potential lipid biomarkers and enhance future glioma therapeutics.

## 1. Introduction

Brain cancer is one of the incurable tumour and devastating malignancies, with poor prognosis and adverse impact on quality of life, particularly on the patient’s cognitive abilities [1]. Glioma is one of the most biologically aggressive, complex, heterogeneous ranges of brain cancers affecting millions worldwide [2,3]. Gliomas are classified from grade I to IV according to the histology of the glial cells, morphology and malignant behaviour of the tumour, and tumours’ molecular information [4]. Histopathology relies mainly on morphological and cytologic characteristics resulting from staining methods. However, brain cancers exhibit wide molecular variability and instability, which remain invisible to microscope-based pathology [5]. Brain tumours’ complexities and variable biological characteristics lead to different treatment outcomes and patients’ survival. Hence, it is imperative to identify endogenous biomarkers for glioma malignancy and determine potential targets for developing effective therapies. To improve the current diagnosis and develop more effective treatments, research in the past few decades focused on investigating molecular aberration in the genome, transcriptomic, proteome and more recently in the metabolome as well as lipidome [6].

Lipids are a group of hydrophobic molecules, composed of a diverse group of lipid compounds. Alteration in lipid metabolism is among cancer’s most major metabolic alterations [7,8,9]. Cancer cells, including glioma, can shift to lipid metabolism as various lipogenic enzymes were reported to be upregulated or activated [10]. Cancer cells manipulate different approaches to acquiring lipids and extensively alter their metabolism driven by both oncogenic and environmental factors to survive and thrive in a changing microenvironment [11]. Multiple preclinical studies described that aberration in lipid metabolism is lipid alteration in glioma, where glioma cells express an increased level of total lipid content compared to normal tissues [12,13,14]. Glioblastoma tissues contain elevated free fatty acyl, long-chain polyunsaturated fatty acids (LC-PUFAs), and different phospholipid compositions compared to normal brain tissue [12]. In addition, severe dysregulation in phospholipid components has been reported in the isocitrate dehydrogenase (IDH) mutation subtype [15]. However, a specific type of lipids has adverse effects on cancer development. For example, docosahexaenoic acid (DHA) and vitamin D regulate cytotoxic effects in tumour cells [16,17].

This systematic review has summarised the significant findings on lipid dysregulation in different glioma models. Knowledge will be further categorised according to their agonists and antagonists’ roles in gliomagenesis. The authors also investigate the prominent lipid species characterised by molecular and lipid metabolic imaging. Importantly, this systematic review will further highlight the individual lipid metabolites that could be identified as potential lipid biomarkers for the clinical benefit of glioma.

## 2. Materials and Methods

This systematic review was conducted according to the preferred reporting items for systematic reviews and meta-analyses (PRISMA) guidelines [18]. The study protocol was registered in the International Prospective Register of Systematic Reviews (PROSPERO) database (CRD42022374750). The study was conducted in three systematic searches to widen the search outcomes. The first systematic search was to employ current lipid classes according to The Lipid Metabolites and Pathways Strategy (LIPID MAPS) consortium. The second search was to identify studies of lipids alteration in glioma; the third was to determine any glioma studies on lipid alteration using imaging approaches. The search algorithm for all three clusters was listed in Supplementary Table S1. The relevant articles were retrieved from 2011 to 2021. Duplicate articles were removed. Potential articles were further evaluated by reading their full texts.

Extensive inclusion criteria were employed in this study due to the inadequate quantity of studies related to glioma and lipid biomarkers. Inclusion criteria of this study are as follow: (1) article type includes research articles, comparative studies, and multicentre study (2) glioma as disease studied (3) study population include human, animal and in vitro experiments, (4) no restriction on analytical techniques employed (5) no age limit in the human population.

Exclusion criteria: (1) article formats including reviews, conference abstracts, comments, letters, meta-analysis and clinical trial studies (2) brain disease other than glioma (3) no suitable control groups were used (4) other languages than English (5) non-traceable and no full-text articles. 

Risk of bias assessments and study quality were conducted using the Newcastle-Ottawa Scale (NOS). The star’s rating system has evaluated three categories: selection, comparability and outcome. The scores of NOS ranged from 0 stars (lowest score) to 9 stars (highest score). A study with a NOS score higher than 5 was recognised as a high-quality study [19].

The data extraction form was performed using Excel Microsoft software. All the essential information includes the first author, year of publication, sample population, analytical modalities, experimental approaches and any changes in lipid metabolites. KR conducted the data search and extraction step, further assessed by another three independent investigators (KR, JHDW, NR), with disagreements resolved by discussion and consensus. Articles were separated for the synthesis into two categories: lipid metabolite and lipid imaging. All the lipid categories, annotation and classification, were standardised according to the LIPID MAP consortium guidelines.

## 3. Results

### 3.1. Eligible Studies

**Figure 1:**
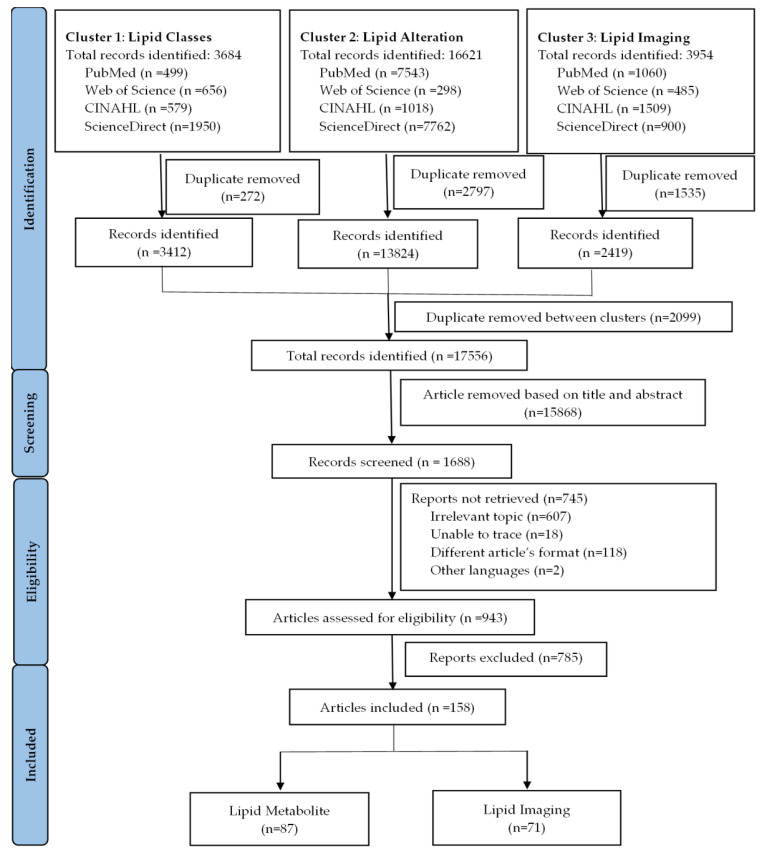
Flow diagram of study selection in the systematic review.

The functional flow diagram is illustrated in Figure 1. A total of 17,556 research articles were identified from four databases by the initial search strategy. 1688 research articles remained for screened after articles were excluded based on the title and abstract. Further selection limits the number of research articles to 943, after exclusion for various reasons, including unrelated content, inability to trace, different article formats and languages. 943 research articles were reviewed, resulting in 158 research articles included in this systematic review. All the research articles included in this systematic review have NOS scores of more than 5, except for 1 research article, which was excluded from further analysis (Supplementary Table S2).

### 3.2. Characteristics of Included Studies

**Figure 2:**
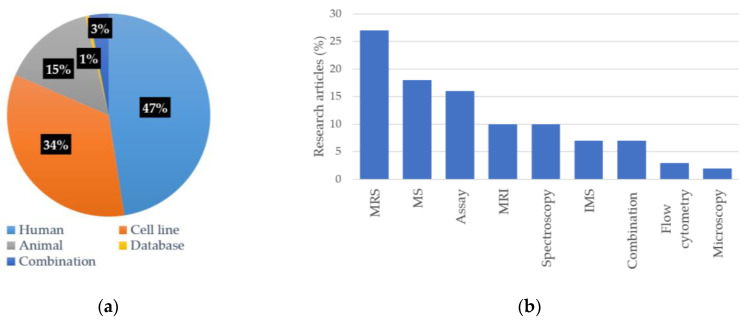
Number of research articles is based on: (**a**) different biological specimens; (**b**) various analytical techniques.

**Figure 3:**
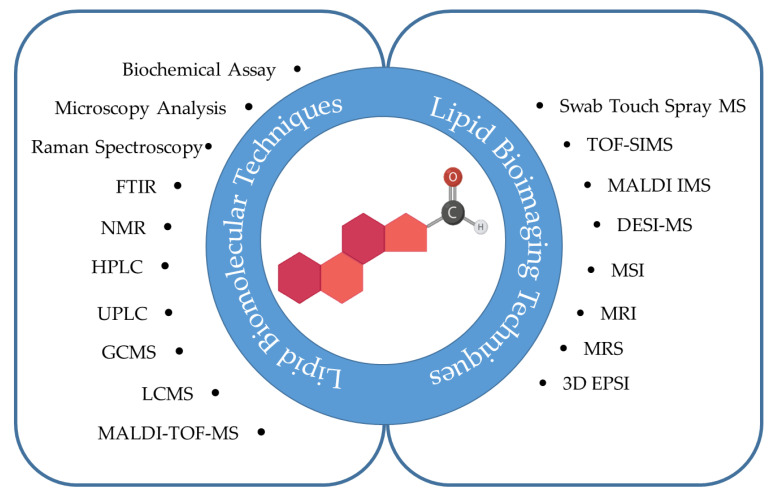
Various analytical techniques were utilised in the included research papers to analyse lipids in glioma. DESI-MS desorption electrospray ionisation mass spectrometry; FTIR Fourier transform infrared; GCMS gas chromatography mass spectrometry; HPLC high-performance liquid chromatography; LCMS liquid chromatography mass spectrometry; MALDI IMS Matrix-assisted laser desorption ionization imaging mass spectrometry; MALDI-TOF-MS Matrix-assisted laser desorption ioniza-tion time-of-flight mass spectrometry; MS mass spectrometry; MRI magnetic resonance imaging; MRS magnetic resonance spectroscopy; MSI mass spectrometry imaging; NMR nuclear Magnetic Resonance; TOF-SIMS time-of-flight secondary ion mass spectrometry; UPLC ultra-performance liquid chromatography; 3D EPSI 3D echo-planar spectroscopic imaging.

The main characteristics of included studies are described in Figure 2. The findings were grouped into two study areas: lipid metabolites and lipid imaging, with 87 and 71 articles, respectively. Different experimental designs were observed in the accumulated research studies, as shown in Figure 2a,b and Figure 3.

### 3.3. Lipids Metabolites Alteration in Glioma

**Figure 4:**
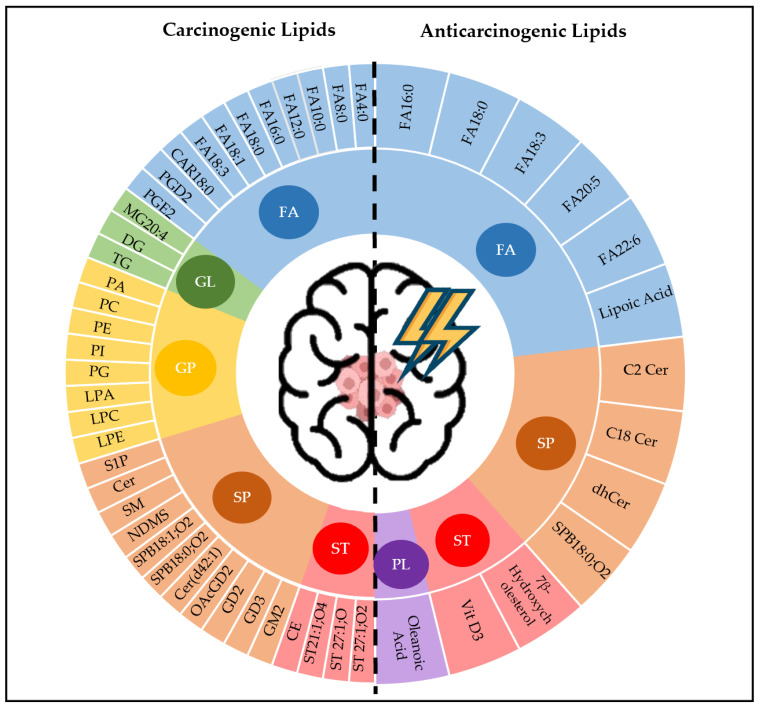
A collective of prominent lipids metabolite from lipid classes with properties of carcinogenic and anticarcinogenic roles in glioma. (Refer to Supplementary Table S3 for the abbreviation).

Overall, 57 lipid classes from 6 lipid categories were detected in glioma. Of these, 42 lipid classes serve supportive roles, while 15 serve suppressive roles in glioma. Out of 8 lipid groups classification, only 6 groups were identified in the included studies. They were fatty acyl (FA), glycerolipid (GL), glycerophospholipid (GP), sphingolipid (SP), sterol lipid (ST), and prenol lipid (PL). No article was found regarding the roles of saccharolipids and polyketides in glioma. Subsequently, identified lipids species were grouped according to their impact on tumourigenesis (Figure 4). Lipids have been recognised to exert various biochemical functions, where some lipids support while some oppress tumour development. Lipidomic data from the systematic literature identified a subset of carcinogenic and anticarcinogenic lipids in glioma (Table 1 and Table 2). Chemical structures for lipid species were enlisted in Supplement Table S4.

#### 3.3.1. Carcinogenic Lipids in Glioma

Twelve studies reported the carcinogenic effects and functions of fatty acyls on glioma. Fatty acyls are generally classified into short-chain fatty acid (SCFA), medium-chain fatty acid (MCFA) and long-chain fatty acid (LCFA). However, only MCFA and LCFA were found in gliomas. The predominant FA reported upregulated in gliomas range from C8 to C20. In contrast, Lauric acid (C12:0) and very long chain dicarboxylic acids (VLCDCA) were reported to be downregulated [22,27]. Glioma cells required constant and excessive energy supply due to active proliferation [20,28]. Besides glucose, ketone bodies are also the preferred energy source [21]. Linolenic acid (C18:3), an LCFA is significantly enriched in the serum of glioblastoma (GBM) patients [20]. Palmitic acid (C16) and octadecanoic acid (C18:0) were enriched in tissue and serum of GBM, respectively [20,23]. Linolenic acid (C18:3) was significantly enriched in the serum of GBM patients [20]. Raman spectroscopy on cell culture showed that increased MCFA such as oleic acid (C18) in glioma, is associated with cellular apoptosis. In contrast, decreased levels of VLCDCA are associated with anti-inflammatory and chemo-preventative properties. Glioma cells have an abundance of PGD2 and PGE2 in their lipidome, and it was hypothesised that the relative increased in these lipids may be associated with treatments resistance potential [29,30,31].

Abnormal glycerolipid metabolism is another pathway that has been commonly reported in glioma. Concentrations of diacylglyceride were different across the studies included, where increased [33] and decreased [21,34] levels were reported. Enhanced expression of DG was associated with malignancy transformation, while reduction of DG particularly DG34:0, DG34:1, DG36:1, DG38:4, DG38:6 and DG4:6 were corresponding to tumourigenesis signalling and inflammatory response in GBM. Additionally, TG was highly utilised by GBM tissue for energy production [35,36]. However, Anna et al. reported that level of TG was significantly increased in medulloblastoma tissue [37]. Further, a brain 2-AG was elevated in both LGG and HGG of human tissue [32].

GP was extensively studied, as 24 research papers reported the detrimental effect of GP on glioma. PA was highly expressed in GBM tissue, which is associated with the lipid signalling towards autophagy mechanism. Thus, prolonged the survival of GBM [38,39]. However, Anna et al. [37] and Wildburger et al. [40] discovered similar lipid signatures with a notable decrease of PA36:2, PA42:5 and PA42.7 in animal and human GBM tissue. Several studies focus on the investigation of the impact of PC in glioma [43,44,48] Enhanced production of PC was reported in astrocytoma and GBM. PC were reported to be responsible for stimulating cell division, tumour progression and malignancy [28,34,41,42,43,44,45,46,47,48]. In addition, the other GP metabolites including PI PG and PE were all highly expressed in HGG [26,33,34,48,53]. Their production was associated with the tumour growth and infiltration. Conversely, level of LPA, LPC and LPE were significantly reduced in both LGG and HGG [54,55,56].

Ceramide is the central metabolite for sphingolipid metabolism [66]. High ceramide production is associated with tumour proliferation. Ceramide is further synthesis to produce sphingomyelin (SM) [98]. Enhanced level of SM was reported in HGG [33,66,67]. S1P is the sphingolipid derivative, derived from ceramide metabolism [66]. S1P is the most lipid studied in glioma, with eleven research papers reported carcinogenic effect in both cell line and human tissues [57,58]. Most of these papers found that high S1P result in resistant to treatment and induced cell growth, proliferation and angiogenesis [57,58,59,60,61,62,63,64,65,66,67]. Other sphingolipid mediators, including sphingosine, NDMS, N-lignoceroylsphingosine were highly expressed in the cell line and tissue of LGG and HGG [28,34,68]. In addition, ganglioside composition was altered in glioma. Distinctive changes in different ganglioside expression such as O-acetyl GD2, GD2, GD3, GM2 and GM3 reflecting a tumour malignancy, migration, and progression in LGG and HGG [70,71,72,73,74,75,76]. O-acetyl GD2 was identified in the human glioblastoma tissue specimen, as it was suggested to induce GBM cell proliferation [70]. Distinctive changes in ganglioside level, particularly GD3, GM1, GD1 and GM3 lipid species [72]. Fabris et al. identified the composition of ganglioside where a mixture of diverse ceramide composition with fatty acyl ranging from 16C to 24C atom was highly prominent in glioblastoma [73], whereas anaplastic and diffuse midline glioma contain notable amount of GD3 (d18:1/24:0) and GD2, respectively in their tissues [74].

GBM tissue was reported to produce excessive amount of cholesterol to support their growth and proliferation [78,79]. Im et al. investigated the changes in CSF lipid profile of grade III glioma. There was an extensive increased level of 1-oleyl cholesterol and tetrahydrocorticosterone, which the authors suggested could be due to the malignancy transformation [33]. Moreover, serum of GBM patient contain high low-density lipoprotein (LDL) that is associated with tumour growth and proliferation [80].

#### 3.3.2. Anti-Carcinogenic Lipids in Glioma

There were 18 articles investigated the beneficial effects of lipid species as anti-carcinogenic in glioma. Palmitic acid and stearic acid have been shown to promote anti-proliferative by increasing activity of neurotoxicity and gliomatoxicity in GBM cell line [81]. Studies by Anta et al. and Antal et al. presented the anticarcinonegic effect of GLA in glioma and GBM cell lines [82,83]. Additionally, treatment of EPA to the GBM cell line resulted in ceased growth of glioma cells [23]. Several studies evaluated the beneficial effect of DHA in glioma. DHA was proposed to possess as anticarcinogenic lipids by reducing therapeutic resistance, preventing tumour migration, preserving the structure of lipid domain located in plasma membrane and inhibit the formation of lipid droplet [82,84,85,86]. The presence of lipoic acid could prevent glioma growth by reducing cell proliferation and increase cell susceptibility towards treatments [82,87].

Many sphingolipids have shown promising results as cancer treatments. Jung et al. found that short carbon ceramide (C2 ceramide) has a positive impact on inhibition of glioma invasion in GBM cell line [88]. Treatment of C18 ceramide also resulted in growth inhibition of in vitro GBM cell line [89]. Moreover, dihydroceramide and dihydrosphingosine produce remarkable effect on GBM cell lines. These compounds work by increasing oxidative stress and further contributing to glioma cell death [90]. Glycosides also exhibit excellent anticancer properties by inducing endoplasmic reticulum stress and increased the rate of apoptosis [91].

A few sterol lipids possess the beneficial effects on the development of glioma. Clarion et al. suggested that 7B-hydroxycholesterol exhibit anticancer property in GBM by reducing composition of cholesterol and its derivative [92]. Many studies have been conducted on the effect of vitamin D3 in GBM cell lines. The results demonstrated that reduction in tumour growth and proliferation in GBM [87,93,94,95]. Sterol lipid derivative, steroidal meleimides have been found to ceased tumour growth and greatly cytotoxic to tumour cells [96]. Additionally, treatment of GBM cell line with oleanoic acid, constituent of prenol lipid causes reduction in tumour cells migration and invasion [97].

### 3.4. Lipids Signal Intensities in Glioma

Raman technology can detect variance related to DNA/RNA, proteins and lipids, have made it an essential tool for examining changes on the cellular level, and generating cell fingerprints of specific diseases [99]. In this systematic review, some studies have documented the reduction in lipid signals, while some authors reported that elevated lipid signals under various experimental conditions. Decreased lipid signals, particularly at 1450 cm^−1^ were found in glioma U251 cell culture and human GBM [37,100,101]. However, high lipid signals were detected in astrocytoma grade IV, recurrent glioma and after temozolomide treatment [102,103,104]. Uckermann et al. also provided similar result where elevated lipid signals were detected by FTIR spectroscopy [105].

Changes in lipid signal intensity have been observed in MRS of different glioma tumours (Table 3). Increased lipid intensity was observed in majority of the studies [106,107,108,109,110,111,112]. However, lipid intensity was reduced in LGG [113,114]. At peak 0.9 ppm, reduction of lipid peak was observed in patient of pilocytic astrocytoma [115], while intense lipid signals were observed in high grade glioma [116,117,118]. Abnormally high lipid signal at 1.3 ppm was observed in GBM by multiple studies [116,117,118,119,120]. Work by Delgado-Goñi et al. also reported high lipid peak at 2.8 ppm [116].

MRS is also useful for detection of choline metabolites. Elevated level of choline was identified in children glioma, including medulloblastoma, optic pathway glioma and grade III glioma [106,121,122]. Similar result was also reported in adult LGG and HGG [118,123,124,125,126,127,128,129,130,131]. Conversely, decreased of choline signals were detected by several studies [113,132,133,134]. Nevertheless, GPC was also consistently increased in glioma [115,118,120,121,127,128].

**Table 3:** Lipid MRS characteristics as detected in the form of signal loss ratio (SLR), lipid fraction, and lipid spectroscopic signals of different glioma tumours.

Reference	Children/Adult	Histopathological Type	Lipid Metabolites Detected					
			Lipid	Lip0.9	Lip1.3	Lip2.8	Cho	GPC
[106]	Children	Medulloblastoma	↑				↑	
[135]	Children	LGG HGG						
[121]	Children	Optic pathway glioma					↑	↑
[122]	Children	Glioma (Grade III)					↑	
[107]	Children	HGG	↑					
[115]	Children	Pilocytic		↓	↓			↑
[108,109,110,111,112,136,137,138]	Adult	GBM	↑					
[123,124]	Adult	GBM	↑				↑	
[132]	Adult	GBM	↑				↓	
[114]	Adult	LGG HGG	↓ ↑					
[120]	Adult (Rat)	GBM			↑			↑
[127]	Adult	GBM					↑	↑
[128]	Adult	Grade II Grade III					↑ ↑	↑ ↑
[116]	Adult	GBM	↑	↑	↑	↑		
[129]	Adult	Grade III (Enhancing area) Grade III (Non-enhancing area)	↑				↑	
[133]	Adult	LGG HGG	↑				↓	
[130,131]	Adult	LGG					↑	
[125]	Adult	Fibrillary Astrocytoma Astrocytoma (Grade III) GBM	↑ ↑				↑ ↑ ↑	
[134]	Adult	LGG HGG					↓ ↑	
[139]	Adult	Astrocytoma (Grade III) GBM	↑					
[126]	Adult	Medulloblastoma Haemangioblastoma	↑				↑	
[117]	Adult	GBM	↑	↑	↑			
[113]	Adult	GBM (Pseudoprogression) GBM (Recurrence)	↑ ↓				↓ ↑	
[119]	Adult	GBM	↑		↑			
[118]	Adult	Grade II Grade III Grade IV	↑	↑	↑		↑ ↑	↑ ↑ ↓

The arrow illustrates if a metabolic feature is (↑) elevated or (↓) reduce. Cho choline; GPC glycerophosphatidylcholine; HGG high grade glioma; LGG low grade glio-ma; MRS magnetic resonance spectroscopy.

## 4. Discussion

### 4.1. Carcinogenic Lipids in Glioma

#### 4.1.1. Fatty acyl (FA)

It was widely recognised that FAs metabolism, including FA synthesis, uptake, modification and degradation, are all dysregulated in cancer, to regulate many biological activities to support cancer cells’ needs [140]. Unexceptionally, aberration of FA metabolisms becomes essential in glioma cells [141,142]. Physiological roles of FA include providing substrates for energy production, membrane phospholipid modification and alteration of signal transduction [143]. In cancer cells, their roles exceed their primary roles as FAs were responsible for a range of carcinogenesis processes, such as induction of blood vessel development for tumour angiogenesis and deactivated apoptosis mechanism by regulating pro-apoptotic factor expression [144,145].

Due to active proliferation, glioma cells require constant and energy supply [146]. Glioma cells show an exceptional ability to utilise different FA, ranging from butyric acid, octadecanoic acid, stearic acid, linoleic acid and arachidonic acid, to fulfil exorbitant energy needs [20,21]. Other than glucose, ketone bodies are also the preferred energy source. In mitochondria, fatty acyls are converted to acetyl-CoA via β-oxidation, which can be utilised to produce ketone bodies under glucose starvation conditions [147]. Lauric acid (LAA), a saturated medium-chain fatty acid (MCFA), can also be catabolised to ketone bodies. They have a higher efficacy than long chain fatty acids (LCFAs), as they can penetrate the mitochondrial inter-membrane region directly without using the carnitine shuttle [148]. This could potentially be due to the lauric acid being more preferable as substrate for synthesising adenosine triphosphate (ATP) in mitochondria; thus, LAA level was reduced in glioma. Unlike lauric acid, octanoic acid (C8) and decanoic acid (C10) levels were increased in glioma. 

**Figure 5:**
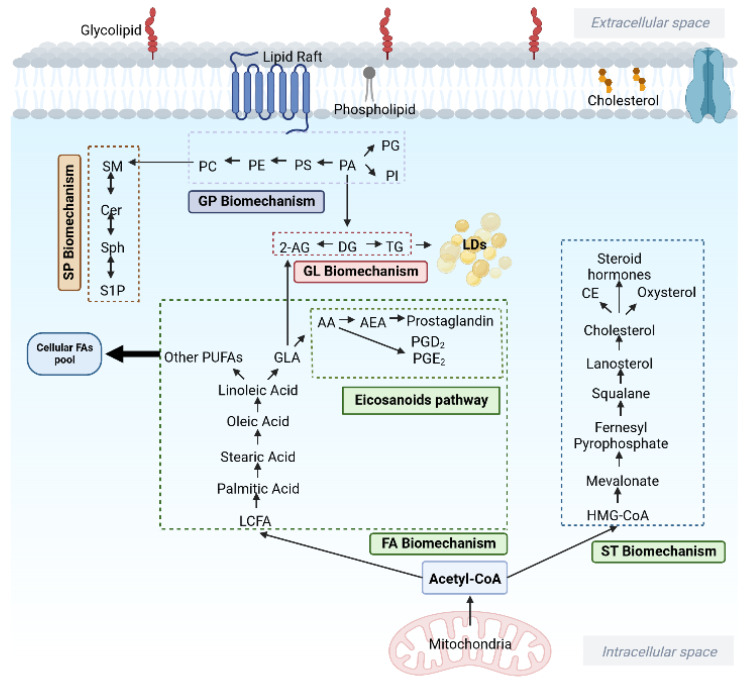
An overview of cellular lipid mechanism, involving lipid biosynthesis from different lipid classes. Adapted from “Phospholipid Bilayer Membrane”, by BioRender.com (2022). Retrieved from https://app.biorender.com/biorender-templates (accessed on 9 November 2022). AA arachidonic acid; AEA anandamide; CE cholesterol ester; Cer ceramide; DG diacylglycerol; FA fatty acyl; GL glycerolipid; GP glycerophosphate; GLA gamma linolenic acid; HMG-CoA 3-hydroxy-3-methylglutaryl coenzyme A; LCFA long-chain fatty acyl; LDs lipid droplets; PA phosphatidic acid; PC phosphatidylcholine; PE phosphatidylethanolamine; PG phosphatidyl-glycerol; PGD2 prostaglandin D2; PGE2 prostaglandin E2; PI phosphatidylinositol; PS phospha-tidylserine; PUFAs polyunsaturated fatty acyls; S1P sphingosine-1-phosphate; SM sphingomye-lin; SP sphingolipid; Sph sphingosine; ST sterol lipid; TG triacylglycerol; 2-AG 2-arachidonoylglycerol.

Excess uptake of LCFAs promotes the development of many cancers by interrupting the normal function of poly-adenosine diphosphate (ADP) ribose polymerase (PARPs) [149]. Linolenic acid (C18:3) is an essential element of cell membranes and the precursor of arachidonic acid, which is associated with the proinflammatory response [150]. Levels of linolenic acid were reported to be increased in many cancers and identified as a biomarker for malignant non-small cell lung cancer (NSCLC) [151,152]. Palmitic acid, the most active and fundamental saturated FA, can be further processed into other lipids and lipid mediators as shown in Figure 5 [153].

Pascual et al. reported that palmitic acid significantly induced the metastatic and increased CD36 cell surface expression in glioma [154]. Similar to those finding, Gaston et al, reported that palmitic acid stimulates proliferation in glioma cell culture at the concentration of 50 mmol and 100 mmol. Additionally, palmitic acid triggered cell invasion and migration in gastric and breast cancer [155,156]. Oleic acid (C18) was found to have a pro-tumourigenic role by inducing the formation of lipid droplets (LDs), enhancing cell proliferation and disrupting cellular apoptosis in glioma [24,25,26,157]. Oleic acid had been reported to enhance cervical cancer cell growth by regulating CD36 expression and promoting cancer cell migration and proliferation [158].

Prostaglandins were extensively investigated for their linked with an extended list of adverse health conditions, including cancer, inflammation, arthritis, atherosclerosis and thrombosis [159]. Prostaglandin is a group of bioactive lipids derived from arachidonic acid that was reported to have a tumourigenesis effect on glioma. Irradiated glioma cells synthesise prostaglandin E2 (PGE2) as a feedback mechanism for cell survival and to prevent cell death [30]. This was achieved by activating the ERK1/2 MAPK pathway, which increases self-renewal capacity and increases the resistance to radiation-induced DNA damage [31]. In addition, prostaglandin D2 (PGD2) was reported to have a pro-tumourigenic role that support the growth and invasion of glioma [29].

#### 4.1.2. Glycolipid (GL)

Diacylglycerol (DG) and triacylglycerol (TG) are classified as glycolipids and play crucial roles in glioma proliferation. DG and TG biosynthesis were upregulated in glioma, and rapidly consumed upon glucose reduction [35,160]. Specifically, DG34:0, DG34:1, DG36:1, DG38:4 and DG40:6 were reduced in glioma tissue [40]. Hydrolysis of DG activated the lipid messenger, which further utilised for the production of secretory vesicles [161,162].

TG is a major component of lipid droplets, and a high presence of lipid droplets is associated with cancer progression [35]. Lipid droplets, also known as adiposomes, are the smallest recognised lipid compartments with approximately 20 to 100 μm diameter that resided close to mitochondria [163]. In addition to serving as energy storage through the beta-oxidation process, these inducible organelles play a crucial role in cell signalling. They regulate the formation of inflammation mediators (e.g., eicosanoids) and are involved in the biosynthesis of free-fatty acyl-derived intermediates (e.g., sphingolipid ceramides) that may lead to lipotoxicity [164]. Lipid droplet formation specifically occurs under restricted conditions such as hypoxia and nutrient deprivation, by inducing an increase in lipoprotein uptake in a heparan sulphate proteoglycan-dependent manner [165,166]. Accumulations of lipid droplets has been associated with various cancers, including hepatic cancer, lung cancer, breast cancer and gliomas [167]. 

2-arachidonylglycerol (2-AG) is a distinct group of monoacylglycerol derivatives of arachidonic acid (AA) that can be produced from AA-enriched membrane phospholipids such as phosphatidylinositol (PI) and phosphatidic acid (PA) [168]. 2-AG together with anandamide (AEA), make up the endocannabinoid system (ECS), which is a signalling network involved in several biomechanisms such as neurobehavioural, inflammation and metastatic promotion in breast cancer [169,170]. Elevated 2-AG was also found in tissue samples of both low- and high-grade glioma [171]. 

#### 4.1.3. Glycerophospholipids (GP)

Glycerophospholipids are critical components of the plasma membranes and function in cell signalling activity [172]. Wildburger et al, reported that glioma enriched in phosphatic acid, particularly PA36:2, PA4:5, PA42:5 and PA42:7 [40]. PA is predominantly utilised with endogenous FAs to provide glycerophospholipids continually for membrane production [173]. The accumulated content of PA is associated with a series of changes to cancer cell metabolism. Primarily, PA upregulate several kinases, such as mitogen-activated protein kinase (MAPK), ABL tyrosine kinase 1 (ABL1) or 3-phosphoiositide-dependent protein kinase-1 (PDK1), involved in intracellular stress signalling pathways [174]. These processes are also associated with tumour initiation and progression [175]. Additionally, enhanced production of PA contributes to the activation of hypoxia-inducible factor 1-alpha (HIF1A) transcription that stimulates angiogenesis and cancer cell proliferation [176]. Furthermore, inhibition of PA biosynthesis was selectively toxic to glioma but not in the normal cells [38]. Lysophosphatidic acid (LPA), which was derived from phosphatidic acid, was increasingly expressed to disrupt the formation of primary cilia in human astrocytes leading to the proliferation of glioma [54]. 

Metabolites of GP: phosphatidylcholine (PC), phosphatidylethanolamine (PE), phosphatidylinositol (PI) and phosphatidylglycerol (PG) were highly intensified in human tissue and cell line model of grade III and GBM. Similarly, the concentration of PC and PE were also elevated with increasing breast cancer grade, indicating that the glycerophospholipid synthesis rate increases with oncogenesis and tumour progression [177]. PC and PE comprised the most phospholipid content in human cell membranes [178,179]. In addition to regulating the plasma membrane, PE acts as lipid chaperone that aids in folding of particular membrane proteins and plays a central role in the initiating autophagy [179]. Ratio of PC and PE composition within the plasma membrane is crucial for imposing curvature stress onto the membrane, that controlled cell trafficking [180]. Moreover, PI contributes significantly to cancer cells by regulating numerous cellular activities involving cell adhesion, migration, apoptosis, and vesicle trafficking to post-translational modifications [181].

#### 4.1.4. Sphingolipid (SP)

The sphingolipid metabolism, particularly sphingosine-1-phosphate (S1P), was extensively studied in glioma. Oancea-Castillo et al. showed that S1P protect glioma cells from radiation and temozolomide treatment [59]. S1P was highly expressed in glioma [60]. Similar results were obtained by Abuhusain and coworkers [61], where S1P content was detected 9-fold higher than normal brain tissue. In the isocitrate dehydrogenase mutation (IDHmut) glioma cells, S1P contents were upregulated, while other sphingolipid such as N,N-demthylsphingosine (NDMS), C17 sphingosine and C18 sphingosine were downregulated [68]. Bernhart et al. showed that the glioma consumed S1P, ceramide and sphingomyelin (SM) in cell studies [66]. Exogenous addition of S1P caused increased glioma proliferation. Apart from the roles in angiogenesis and tumour growth, S1P also decreases glioma cells’ radiosensitivity to temozolomide and other treatments [62]. These data suggested that the crucial role of S1P in the progression and resistance to treatment.

The elevated level of ceramide in cerebrospinal fluid (CSF) and tissue samples in grade III glioma and GBM, increased massively. Ceramide is a product of the sphingomyelinase-catalysed mechanism, triggers cytochrome c transported out from mitochondria and subsequently activates the apoptotic pathway in tumour cells [182]. Alternatively, ceramide is utilised as a substrate to produce glucosylceramide (GlcCer), SM and S1P, leadings to lipid abnormalities in the membrane [183]. This explained the high concentration of ceramide to support the high demand for sphingolipid in glioma cells. Additionally, elevated composition of endogenous ceramide could potentially activate JNK and p38 signalling in the surrounding cells to promote cellular apoptosis [184].

Ganglioside is a group of acidic glycosphingolipids (GSLs) abundant in outer leaflet of cell membranes, specifically enriched in lipid microdomains and neuronal cell [185]. GSLs together with sphingolipid and cholesterol is the constituent of lipid rafts [186]. Lipid raft plays an essential role in the biosynthetic and endocytic vesicular trafficking, ceramide-mediated apoptosis, host-pathogen communications (protein binding and uptake), cytoskeletal dynamics and rearrangement, cellular polarisation and regulating signal transduction [187]. Disassembles of lipid raft in the plasma membrane will further activate diverse signalling cascades that stimulate cell survival, proliferation, differentiation, migration and invasion in cancers [188]. Moreover, ganglioside lipid was present in glioma cells while absent in normal brain tissue [76]. GD2 is not frequently secreted in normal brain tissue but is overexpressed across different types of tumours, particularly those with neuroectodermal origins such as neuroblastoma melanoma and small-cell lung cancer [189,190]. In general, different types of gangliosides are released by cancer cells that are important for angiogenesis in tumour [191]. Notably, changes in gangliosides were highly expressed in various types of cancer, such as hepatocellular carcinoma, pancreatic cancer, glioma and skin cancer [192].

#### 4.1.5. Sterol Lipids (ST)

Sterol lipid is a diverse group of lipid categories with four ring structures [193]. Sterol lipids play a crucial role as the main component of cellular membranes and regulation of membrane fluidity. However, sterol lipids are also associated several disorders, including cancer [194]. Patel et al. stated that the expression of cholesterol in tumour specimens was associated with poor survival in GBM patients [195]. Their findings concur with Li et al. where cholesterol level was associated with the tumour grade in glioma [196]. High-grade glioma (HGG) has higher activated cholesterol biosynthesis relative to the low-grade glioma (LGG) [197]. Dysregulation of cholesterol leads to the formation of cholesterol ester (CE), which was distinctly different between GBM, LGG and normal brain tissues [77]. Excess cholesterol is converted and stored in the form of cholesterol ester (CE) by acyl-CoA cholesterol acyltransferase (ACAT) [198]. HGG produced higher CE compared to the LGG, while absence in healthy brain tissue [77]. 

24S-hydroxycholesterol, also known as cerebrosterol is responsible for transporting excessive cholesterol to the liver for further degradation to bile acids [199]. Decreased level of 24S-OHC were detected in GBM samples and is associated with the tumour malignancy. These might be a feedback mechanism to remain high cholesterol level for the survival of glioma cells [200].

Sterol lipids play crucial roles in carcinogenesis [201]. Aberration in cholesterol homeostasis may lead to excess cholesterol levels in the brains. Potential sources of additional cholesterol are likely from these processes: excessive production of glial cells, plasma membrane degradation, myelin breakdown and neuronal loss [202]. In multiple studies, cholesterol and cholesterol derivatives were elevated in grade III and GBM [33,77,78,79,80]. Asides from the enhanced intracellular cholesterol level in human tissue, a relative increase in cholesterol levels in blood serum were also observed. Cholesterol content contributes to the membrane fluidity regulation, which modulates the chemotherapeutic resistance and metastatic progression in cancer cells [197]. Tumour cells with elevated cholesterol concentration exhibited higher drug resistance than those with lower cholesterol concentrations [203]. 

Aberrant cholesterol metabolism also increases 24S-hydroxycholesterol (24-OHC) levels in GBM cell culture. The presence of the hydroxy group in 24-OHC make the compound more lipophilic and enables it to transport across the blood brain barrier (BBB) directly [202]. Increased concentration of 24-OHC is associated with the progressive deterioration of myelination [204]. 

Steroids, particularly tetrahydrocorticosterone, are highly accumulated in CSF patients with grade III glioma. However, molecular mechanisms underlying the impact of corticosteroids on tumour cell proliferation are still poorly understood [205]. While several cancers such as colon, lung, cervix, breast cancer and leukaemia, were associated with low serum cholesterol. Brain cancer was associated with elevated levels of plasma cholesterol [206]. 

### 4.2. Anti-Carcinogenic Lipids in Glioma

#### 4.2.1. Fatty Acyls (FA)

Extensive studies have shown that PUFAs have anti-cancer properties and has been demonstrated to produce beneficial effect against glioma under both in vitro and in vivo set-ups [82,83,207,208]. Oleic acid was among the most studied in the included articles. Oleic acid has been shown to reduce polar lipid species and increase radiosensitivity of glioma cells toward radiotherapy treatment [82,208,209]. Palmitic acid (C16) saturated fatty acyl causes depletion in lipid droplets, significantly lowering glioma cells’ survival [86]. In addition, palmitic acid has neurotoxicity effects by increasing oxidative stress and further causing glioma cell apoptosis [81,210]. Accumulating palmitic acids in the cell can stimulate autophagy, a mechanism of cell death via activating the mammalian target of rapamycin (mTOR)-independent signalling pathway [211].

Docosahexaenoic acid (DHA), an omega-3 unsaturated fatty acyl, was reported to have detrimental effects on glioma. Lipid levels in glioma were dramatically decreased when DHA was introduced into GBM cell lines [40,84]. DHA also has a protective role on the plasma membrane by preserving the order of lipid raft [85]. Experimental studies proved that gamma-linolenic acid (GLA), an n-6 fatty acyl exhibits anti-tumoural activity against glioma and GBM cell culture [82,83]. Previous in vivo and clinical investigations have demonstrated the effectiveness of GLA in reducing tumour growth in the rat and human glioma models [173]. In the normal brain, eicosapentaenoic acid (EPA) and DHA were found to provide beneficial effects on neurite outgrowth and synaptogenesis in different cell types and stages of development [212]. In glioma and GBM cell culture, EPA could cease tumour growth and proliferation [156]. 

Lipoic acid induces radio sensitivity in glioma cell culture, which makes the tumour cell more susceptible to treatments. Interestingly, lipoic acid has been demonstrated to induce hypermethylation of the O^6^-methylguanine-DNA methyl-transferase (MGMT) promoter, leading to decreased MGMT proteins in GBM [213]. The result supported that lipoic acid exerts detrimental effects on glioma cells as hypomethylation of MGMT is directly associated with the high resistance toward temozolomide chemotherapy treatment. Furthermore, work done by Deveci et al. concluded that lipoic acid stimulated anti-apoptotic, antioxidant and anti-inflammatory responses via the suppression of the transient receptor potential vanilloid 1 (TRPA1) pathway in glioma cell culture [214].

#### 4.2.2. Sphingolipid (SP)

Ceramide was associated with apoptosis induction in glioma cells, particularly, C2-ceramide, C6-ceramide, C18-ceramide and a mixture of long-chain [63,64,89]. Studies were done by Jung et al. specifically detected C2 ceramide in glioma cells. The finding reported that C2 ceramide suppressed matrix metalloproteinase (MMP) expression and inhibited glioma invasion [88]. Sphingomyelin is structurally similar to phosphatidylcholine but composed of N-acylsphingosine (ceramide). An elevated level of sphingomyelin was detected in glioma tissue compared to the control group [69]. 

Romero-Ramirez et al.’s data highlighted sphingomyelin’s protective role against oxidative stress [91]. In addition, ganglioside was also identified in the included articles. 

#### 4.2.3. Sterol Lipids (ST)

The active configuration of vitamin D (1α,25-dihydroxyvitamin D) functions as a steroid hormone and binds to the vitamin D receptor. This receptor is expressed in most cells and tissues, including neuronal and glial cells in the central nervous system [215]. In addition, Vitamin D has the ability to regulate gene expression in most cell types. Vitamin D has been reported to affect cell proliferation and differentiation, influences the immune system as well as regulates hormone homeostasis [93]. In vitro investigations have been observed that vitamin D3 prevent cell proliferation and triggers apoptosis in several tumour cell lines, such as breast, prostate, colon and ovarian cancer cell lines [216,217]. Vitamin D3 derivatives, including calcipotriol and tacalcitol were found to be beneficial against glioma. Calcipotriol mainly inhibit tumour proliferation by reducing the migration rate of glioma cells [93]. 

Steroids contain a group of molecules derived from cholesterol and play various biological activities. In humans, they may exert beneficial or detrimental impacts on health depending on the type of steroid and/or their derivatives [93,218]. Several maleimides derivatives, such as granulatimide are mainly identified as potential inhibitor exhibiting high antitumour activity [219]. Moreover, natural products comphorataanhydride A and camphorataimide B containing maleimide unit, were shown to have appreciable cytotoxic effects on Lewis lung carcinoma cell lines [220]. From Table 2, we identified that steroidal maleimides produced a cytotoxic effect on glioma cell culture. These findings suggest that vitamin D and steroidal maleimide have potential applications in glioma prevention and treatment [87,93,94,95,96].

#### 4.2.4. Prenol Lipid (PR)

Oleanolic acid (OA), a triterpenoid natural compound contained in various plants, fruits and herbs [221]. OA possesses anti-inflammatory properties and inhibits many malignancy activities in glioma cells [222]. Importantly, OA produces no cytotoxicity in normal human cells [221]. These suppression effects of OA are due to its silencing of some specific intracellular signalling pathways, such as signal transducer and activator of transcription 3 (STAT3), c-Jun N-terminal kinase (JNK), Ak strain transforming (Akt) and nuclear factor kappa B (NF-kappaB) signalling pathways [222,223]. Similar to the finding by these studies, OA causes decreased tumour cell migration and invasion in GBM cell culture.

### 4.3. Lipid Metabolites Alteration on MRS

Lipid metabolic alteration in glioma can be further visualised by non-invasive metabolic imaging [224,225]. Magnetic resonance spectroscopy (MRS) was the primary choice of modality to quantify lipids in glioma (Figure 2). MRS is a non-invasive technique that provides molecular imaging of tissue and is commonly used to examine metabolic alterations that are associated with tumour activity and tumour-tissue characteristics in brain tumours [226]. Table 3 summarised lipid and lipid metabolites signals by using MRS. 

#### 4.3.1. Choline

The metabolic profile most frequently detected in brain tumours includes increased choline (Cho), decreased N-acetyl-aspartate (NAA) and the presence of lactate and lipids [227]. High-resolution 1H-MRS capable of resolving the signals from the individual components of the total choline (tCho) signal, confirming the increase of PC in multiple cancers such as brain, breast and prostate [228,229,230]. An increased tCho signal, which consists of signals from PC, GPC and free choline (Cho) has been detected in various cancers [231].

Choline peaks were the second most detected, where studies reported high levels of choline. Choline is an essential precursor of the Kennedy pathway, responsible for the production of phospholipids in the cell membranes [232]. Choline is phosphorylated by choline kinase (CK) to produce phosphatidylcholine (PC). The presence of choline peaks reflects the elevated cell membrane synthesis and thus increased cellularity [233]. Some authors found the reduction in the PC for LGG, and most of the studies agreed that high levels of PC were detected in GBM. Huang et al., 2010 suggested that high choline was an accumulation effect of overexpression of CK and activation of the transcription factor, including hypoxia-inducible factor-1 (HIF-1) and vascular endothelial growth factor (VEGF) [234].

#### 4.3.2. Lipids Signal

High lipid peaks were detected in both children and adult glioma. Similar results were obtained in this systematic review, where all of the studies included showed changes in the lipids and their metabolites, as reported in Table 3. In brain tumours, the presence of lipids generally indicates the presence of necrotic tissue, which is suggested to be an indicator of malignancy, amount of necrosis as well as a poor prognosis [139,235]. Furthermore, prominent lipid peaks were present in HGG compared to LGG. The most prominent lipid peaks were 0.9 ppm and 1.3 ppm due to the resonance arising from methylene (-CH₂-CH₂-CH₂-) and methyl (CH₃-CH₂-) groups respectively [236].

In normal physiology, lipid signals are considerably tiny, and pathological events such as necrosis or apoptosis will substantially increase lipids. When a high signal appear between 0.9 and 1.3 ppm, their most likely assignment is from methylene and methyl groups of mobile saturated lipids, possibly increasing as an outcome of cell membrane degradation [237]. Moreover, the lipid peaks identified in most pathological processes are predominantly saturated lipids caused by the generation of cytoplasmic vesicles, especially in the necrosis and inflammation regions [238]. The presence of lipids also correlates with the elevated proportion of cells in the S and G phases of the cell cycle [239].

Chemical-shift-based magnetic resonance imaging techniques represent another methods that could measure lipids [240]. These imaging methods produces fat-signal fraction as their quantitative endpoint. The fat signal is the ratio of the proton fat signal to the sum of the proton fat and bulk-free water signal [241]. Table 3 shows lipid signals detected in the form of signal loss ratio (SLR), lipid fraction, and lipid spectroscopic signals. All reports provide a similar result that lipid signal was significantly altered in glioma. Lipid signals have also been detected in vivo in human cancers, such as neuroblastoma and adult brain tumours [242,243]. The theory suggests that mobile lipid signals develop from neutral within the plasma membrane [244]. At the same time, some propose that the presence may be due to the accumulation of lipid droplets in the cytoplasm within the tumour or the surrounding necrotic area [245,246]. Moreover, lipid droplet was also accumulated in response to low pH [247], and treatment with chemotherapeutic drugs [248]. 

Seow et al. demonstrated that the lipid signals in non-enhancing region were associated with glioma grades further result in poor survival [249]. Excessive lipid composition in non-enhancing region was the outcome from the damaged blood vessels and may reflect the aggressive behaviour of high-grade glioma [250]. Distinct lipid contents in non-enhancing region will then contribute to the various tumour heterogeneity in glioma, resulting in different responses to treatments [236].

## 5. Conclusions

Glioma is a fatal brain tumour with unique clinical evaluation and molecular characteristics. In recent years, lipid metabolism reprogramming has earned renewed interest in the oncology field, and evidence of lipid remodelling is emerging in regulating cancer reprogramming. This systematic review summarises the accumulated evidence of abnormal lipid metabolism in glioma. Previously, fewer research were conducted on lipid aberration mainly due to the greatly diverse chemical structures and the limitation on analytical instruments. However, this scenario has changed due to the current progress in analytical technologies aided by algorithms as well as improved databases system, enabling the detection of broad coverage of lipid metabolites. Owing to the complexity of the brain cellular metabolism, different glioma model such as tumour cell lines, xenograft mouse models and glioma patients provided vast information on the aberration of lipid metabolism. In addition, various analytical analyses were employed to measure lipid composition in glioma. In this systematic review, we discussed lipid species on basis of lipid metabolite and lipid metabolic imaging, to provide a comprehensive overview on lipid metabolism reprogramming in glioma. 

Metabolic reprogramming is a crucial hallmark of cancer, where cancer cells demonstrated various rewiring in their metabolic activities. Taken together, we found that glioma cells possess a complex array of lipid species including fatty acyls, glycerolipids, glyceophospholipids sphingolipids and sterol lipids. Indeed, targeting carcinogenic lipids is a key step in developing potential therapeutic targets to suppress glioma growth. However, anti-carcinogenic lipids exhibit detrimental effects on glioma, which may be a novel therapeutic strategy to treat glioma. The studies discussed herein defined preliminary panels of tumour-associated lipids that could aid in the glioma management.

In conclusion, this systematic review provides cumulative evidence of lipid metabolism reprogramming in glioma using different experimental models. We deduced that:

(1) Glioma shifted metabolic plasticity; exert lipid metabolic differences producing lipogenic phenotypes.
(2) Paediatric and adult gliomas have distinct lipid molecular profiles, where glycerophospholipids and fatty acids were among the most affected lipid classes.
(3) The highlighted carcinogenic lipids were recognised to provide a favourable environment for glioma cells growth, proliferation, metastases and survival.
(4) Conversely, the anti-carcinogenic lipids offer promising lipids compounds as possible innovative targets to be further investigated and developed as an innovative treatment strategy for glioma.
(5) The advances of emerging in lipid characterisation techniques, both lipid molecular and imaging techniques expand our fundamental knowledge and perception of bioactive lipid metabolite in glioma tumour aetiology.

A deep understanding of lipids dysregulation in glioma may offer new opportunities to develop new drug delivery strategies, allowing more selective targeting of cancer cells, thus improving the quality of cancer therapy in patients. Despite the emerging interest in lipid function in glioma, further work are required for the translation of lipid biomarkers to routine clinical use.

## Figures and Tables

**Table 1:** Carcinogenic roles of lipid in glioma.

Research	Diagnosis	Experimental Model	Sample Type	Analytical Platform	Lipid Species	Physiological Effect on Glioma
Fatty Acyls (FA)						
[20]	GBM	Human	Serum	GC-TOFMS	↑ Butyric acid (C4)	Provide substrate for glutamate metabolism
[21]	GBM	Cell line	Tissue	GCMS	↑ Octanoic acid (C8)	Activated ketone body metabolism for glioma cell survival
[21]	GBM	Cell line	Tissue	GCMS	↑ Decanoic acid (C10)	Stimulate fatty acyl production
[22]	GBM	Human	Tissue	GC-TOFMS	↓ Lauric acid (C12)	Tumour malignancy
[23]	GBM	Cell line	Tissue	HPLC, Spectrophotometric	↑ Palmitic acid (C16)	Enhanced glioma cell proliferation
[20]	GBM	Human	Serum	GC-TOFMS	↑ Stearic acid (C18:0)	Provide substrate for glutamate metabolism
[24,25,26]	Oligodendroglioma Astrocytoma GBM, glioma	Cell line	Tissue	Raman spectroscopy MS	↑ Oleic acid (C18:1)	Cellular apoptosis, Enhance proliferation of GBM cells
[20]	GBM	Human	Serum	GC-TOFMS	↑ Linolenic acid (C18:3)	Provide substrate for glutamate metabolism
[20]	GBM	Human	Serum	GC-TOFMS	↑ AA (C20:4)	Provide substrate for glutamate metabolism
[27]	GBM	Human	Serum	MS	↓ VLCDCA	Anti-inflammatory and has chemopreventative properties
[28]	Glioma (Grade II, III), GBM	Human	Serum	LCMS/MS	↑ Stearoylcarnitine (C18), margaroylcarnitine, Eicosenoylcarnitine (C20:1)	Supply substrate for the activation of fatty acyl metabolism
[29]	GBM	Cell line	Tissue	HPLC-MS/MS	↑ PGD2	Support glioma growth and invasion
[30] [31]	Glioma GBM	Cell line Human & animal	Tissue	Biochemical assay	↑ PGE2	Protect glioma cells against radiation treatment. Escalate self-renewal capacity and resistance to radiation-induced DNA damage
**Glycerolipid (GL)**						
[32]	LGG, HGG	Human	Tissue	LCMS	↑ 2-AG	Inhibit cell apoptosis, support cell proliferation and survival
[33]	Grade III	Human	CSF	MS/MS	↑ DG	Malignant transformation
[21,34]	GBM	Cell line	Tissue	GCMS	↓ DG (DG34:0, DG34:1, DG36:1, DG38:4, DG38:6, DG40:6)	Influence carcinogenesis signaling and inflammatory reaction in GBM
[35,36]	GBM	Cell line	Tissue	Microscopy	↓ TG, LD	Mitochondria hydrolyzed lipid droplets and utilized triglycerides for energy production
[37]	Medulloblastoma	Human	Tissue	Raman Imaging	↑ TG	Tumour development
**Glycerophospholipid (GP)**						
[38,39]	GBM	Cell line	Tissue	Biochemical assay, UPLC-MS/MS	↑ PA	Lipid signaling of autophagy and cell survival in glioma
[37,40]	GBM, glioma	Animal, human	Tissue	MALDI-IMS ESI-MS/MS, Raman imaging	↓ PA (PA36:2, PA42:5, PA42.7) ↓ DHA	Influence carcinogenesis signaling and inflammatory reaction in GBM
[41]	IDHwt glioma	Cell culture	Tissue	NMR	↑ PC	Increase cell turnover and tumour growth
[34,42,43,44,45,46,47] [28,48,49,50,51,52]	GBM Astrocytoma, GBM	Cell line Human	Tissue Tissue, Serum	NMR LCMS	↑ PC ↑ PC PC14:2	Malignant progression and aggressiveness in GBM
[26,34,48] [33]	Glioma Grade III	Human	Tissue CSF	Swab TS-MS, LCMS MS/MS	↑ PI, PG	Enhanced tumour infiltration
[34,53]	GBM	Cell line	Tissue	MSI, LCMS	↑ PE	Enhanced tumour growth
[41]	IDHwt glioma	Cell line	Tissue	NMR	↑ PE	Increase cell turnover and tumour growth
[54]	GBM	Animal	Tissue	MRI	↓ LPA	Support cell proliferation through the disassembling of primary cilia
[55,56]	Glioma Grade II, III GBM GBM	Human Cell line	Tissue	GCMS. LCMS	↓ LPC, LPE	Provide substrates to mitochondria to generate energy
**Sphingolipid (SP)**						
[57,58,59,60,61,62,63,64,65]	GBM	Cell line	Tissue	Biochemical assay	↑ S1P	Resistant to chemo-therapeutic treatment and sustain the growth of glioma cells. Induced cell angiogenesis
[66,67] [33]	GBM Grade III	Cell line Human	Tissue CSF	Biochemical assay MS/MS	↑ S1P, ↑ Cer, ↑ SM	GBM cell proliferation
[68]	Oligodendroglioma	Cell line	Tissue	LCMS	↑ NDMS, ↑ Sphingosine-C18, ↑ Sphingosine C17, ↑ Sphinganine C17	Signaling roles for proliferation and survival
[28,69]	Glioma Grade II-III and GBM	Human	Serum Tissue	LCMS/MS, MALDI-TOF-MS	↑ SM (d16:1/23:0, d17:1/18:0, d18:1/17:0, d18:0/15:0, d18:1/16:0, d18:0/17:0, d19:1/16:0)	Involved in the regulation of sphingolipid metabolism and malignancy of glioma, glioma cell senescence and apoptosis
[33]	Grade III	Human	CSF	HPLC/MS	↑ N-Lignoceroylsphingosine	Involved in lipid signaling and apoptosis
[70]	GBM	Human	Tissue	Biochemical assay	↑ OAcGD2	Increase tumour density and involvement in immunoresistance
[71]	Diffuse midline glioma	Cell line	Tissue	Biochemical assay	↑ GD2	Enhanced metastasis
[72,73,74]	GBM, anaplastic oligodendroglioma	Animal	Tissue	Biochemical assay	↑ GD3	Involved in cell transformation and malignancy
[75]	GBM	Cell line	Tissue	Biochemical assay	↑ GM2	Enhanced cell migration
[76]	Glioma, Medulloblastoma	Animal	Tissue	MALDI-IMS	↑ GM3	Induced malignancy, invasiveness and progression of tumour
**Sterol Lipid (ST)**						
[77]	GBM	Cell line	Tissue	Biochemical assay	↑ CE	Tumour progression and malignant
[33]	Grade III	Human	CSF	HPLC/MS	↑ 1-Oleyl-cholesterol	Enhance tumour growth
[33]	Grade III	Human	CSF	HPLC/MS	↑ Tetrahydrocorticosterone	Enhanced metastasis
[78,79]	GBM	Cell line	Tissue	GCMS Biochemical assay	↑ 24S-OHC	Induced tumour growth by regulating proinflammatory immune cells. nduced pathogenesis of tumour cells
[80]	GBM	Human	Serum	Biochemical assay	LDL	Support proliferation and growth of glioma

↑ denotes increased levels and ↓ denotes decreased levels. AA arachidonic acid; CE cholesterol ester; Cer ceramide; DG diacylglycerol; DHA docosahex-aenoic acid; GBM glioblastoma; GC-TOFMS gas chromatography-time of flight- mass spectrom-etry; GCMS gas chromatography mass spectrometry; GD2 disialoganglioside; GD3 ganglioside GD3; GM2 ganglioside M2; GM3 ganglioside GM3; HGG high grade glioma; HPLC high per-formance liquid chromatography; HPLC/MS high performance liquid chromatography—mass spectrometry; HPLC-MS/MS high performance liquid chromatography with tandem mass spec-trometry; IDHwt isocitrate dehydrogenase (IDH)-wildtype; LCMS liquid chromatography mass spectrometry; LCMS/MS liquid chromatography with tandem mass spectrometry; LD lipid droplet, LDL low density lipoprotein; LGG low grade glioma; LPA lysophosphatidic acid; LPC lysophosphatidylcholine; LPE lysophosphatidylethanolamine; MALDI-IMS matrix-assisted laser desorption ionization—imaging mass spectrometry; MALDI-TOF-MS matrix-assisted laser de-sorption ionization time-of-flight mass spectrometry; MS mass spectrometry; MS/MS tandem mass spectrometry; MRI magnetic resonance imaging; NMR nuclear magnetic resonance; OAcGD2 O-Acetyl-ganglioside 2; PA phosphatidic acid; PC phosphatidylcholine; PE phosphati-dylethanolamine; PG phosphatidylglycerol; PGD2 prostaglandin D2; PGE2 prostaglandin E2; PI phosphatidylinositol; S1P sphingosine-1-phosphate; SM sphingomyelin; Swab TS-MS swab by using touch spray-mass spectrometry; TG triacylglycerol; VLCDCA Very long chain dicarbox-ylic acids; 2-AG 2-arachidonoylglycerol; 24S-OHC 24(S)-hydroxycholesterol.

**Table 2:** Anti-carcinogenic roles of lipid in glioma.

Research	Diagnosis	Experimental Model	Sample Type	Analytical Platform	Lipid Species	Physiological Effect on Glioma
Fatty Acyl (FA)						
[81]	GBM	Cell line	Tissue	Biochemical assay GCMS	Palmitic acid (C16),	Increase activity of neurotoxicity and gliatoxicity in glioma cells.
[81]	GBM	Cell line	Tissue	Biochemical assay GCMS	stearic acid (C18)	Increase activity of neurotoxicity and gliatoxicity in glioma cells.
[82]	Glioma	Cell line	Tissue	Biochemical assay	GLA (C18:3)	Enhanced radio sensitivity towards radiotherapy
[83]	GBM	Cell line	Tissue	MS	GLA (C18:3)	Reduced the number of lipid droplet formation and induced cell death to GBM cells
[23]	GBM	Cell line	Tissue	HPLC, Spectrophotometric	EPA (20:5)	Ceased growth of glioma cells.
[82]	Glioma	Cell line	Tissue	Biochemical assay	DHA (C22:6)	Enhanced radio sensitivity towards radiotherapy
[84]	GBM	Cell line	Tissue	STED microscopy	DHA (C22:6)	Affect the configuration of membrane lipid order, that link to cell migration
[85]	Glioma	Animal	Tissue	Biochemical assay	DHA (C22:6)	Preserve lipid domain in membrane bilayer.
[86]	GBM	Cell line	Tissue	SRS Microscopy	DHA (C22:6)	Decreased the survival rate of glioma cell by reducing the formation of lipid droplets
[87]	GBM	Cell line	Tissue	Biochemical assay	Lipoic acid	Retarded glioma growth by reduced cell proliferation
[82]	Glioma	Cell line	Tissue	Biochemical assay	Lipoic acid	Enhanced radio sensitivity towards radiotherapy
**Sphingolipid (SP)**						
[88]	GBM	Cell line	Tissue	Biochemical assay	C2 ceramide	Prevent glioma invasion through inhibition of MMP expression
[89]	GBM	Cell line	Tissue	Biochemical assay	C18 ceramide	Inhibit cell viability and prevent glioma growth
[90]	GBM	Cell line	Tissue	Biochemical assay	dhCer, dhSph	Increase oxidative stress, endoplasmic reticulum stress autophagy in glioma cells
[91]	GBM	Animal	Tissue	Biochemical assay, LCMS	Glycosides	Induces endoplasmic reticulum stress and cell death
**Sterol Lipid (ST)**						
[92]	GBM	Animal	Tissue	LCMS	7B-hydroxycholesterol	Reduce level of cholesterol, cholesterol ester and cholesterol derivatives
[87,93,94,95]	GBM	Cell line	Tissue	Biochemical assay	Vitamin D3	Reduced tumour growth and prevent proliferation
[96]	GBM	Animal	Tissue	Biochemical assay	Steroidal maleimides	Ceased tumour growth and highly cytotoxic to tumour cells
**Prenol Lipid (PL)**						
[97]	GBM	Cell line	Tissue	Biochemical assay	Oleanoic acid	Reduced tumour cells migration and invasion

DHA docosahexaenoic acid; dhCer dihydroceramide; dhSph dihydrosphingosine; EPA eicosapentaenoic acid; GBM glioblastoma; GCMS gas chromatography mass spectrometry; GLA gamma linolenic acid; HPLC high performance liquid chromatography; LCMS liquid chromatography mass spectrometry; MS mass spectrometry; SRS microscopy stimulated raman scattering microscopy; STED microscopy stimulated emission depletion microscopy.

## Supplementary Materials

The following supporting information can be downloaded at: https://www.mdpi.com/article/10.3390/metabo12121280/s1, Table S1: Search keywords used in the database search; Table S2: Newcastle-Ottawa Scale (NOS) of all researches papers for the assessment of risk of bias; Table S3 List of abbreviations for Figure 4; Table S4 Chemical structures of the studied lipids. The chemical structures and lipid details were acquired from LIPID MAPS, Kyoto Encyclopedia of Genes and Genomes (KEGG), and PubChem databases.
