# Supplementary material for: Lipid Alterations in Glioma: A Systematic Review
Source: Metabolites. 2022 Dec 16;12(12):1280. doi: 10.3390/metabo12121280 (PMC9783089; doi:10.3390/metabo12121280)
Supplement: Supplementary file 1 [file metabolites-12-01280-s001.zip › metabolites-2000074-SI.pdf]

Table S1. Keywords for literature search in databases.

Number	Keyword
Cluster 1	
1	Fatty Acyls
2	Glycerolipids
3	Glycerophospholipids
4	Sphingolipids
5	Sterol lipids
6	Prenol lipids
7	Saccharolipids
8	Polyketides
9	Glioma
Cluster 2	
1	lipid biomarker
2	lipid alteration
3	lipid dysregulation
4	lipid homeostasis
5	lipid metabolism
6	lipid profiling
7	lipid modification
8	lipid balance
9	lipid heterogeneity
10	lipidomic
11	glioma
12	glioblastoma
Cluster 3	
1	Lipid sequence
2	Lipid fraction
3	In- and opposed phase sequence
4	Magnetic resonance spectroscopy
5	Glioma

Table S2. Newcastle-Ottawa Scale (NOS) of all researches papers for the assessment of risk of bias.

Study	Selection	Comparability	Outcome	NOS Score
Lita et al., 2021	★★★	★★	★★	7
Tain et al., 2019	★★★★	★★	★★	8
Nakajima et al., 2019	★★★★	★★	★★	8
Wildburger et al., 2015	★★★	★★	★★★	8
Antal et al., 2014	★★★★	★★	★★	8
Xu et al., 2021	★★★	★	★★	6
Zhu et al., 2015	★★★	★	★★	6
Yuan et al., 2021	★★★	★	★★	6
Ng & Say., 2018	★★★★	★	★★	7
De Oliveira et al., 2018	★★★★	★	★★	7
Damiano et al., 2020	★★★	★	★★	6
Gaston et al., 2017	★★★	★	★★	6
Barcelo-Coblijn et al., 2011	★★★	★	★★	6
Brocard et al., 2014	★★★★	★	★★	7
Cook et al., 2016	★★★★	★	★★★	8

Ferreira et al., 2017	★★★★	★	★★	7
McConnell et al., 2018	★★★★	★	★★	7
Wu et al., 2012	★★★★	★★	★★	8
Garcia-Alvarez et al., 2013	★★★★	★	★★	7
Wu et al., 2020	★★★	★	★★★	7
Bruntz et al., 2013	★★★★	★	★★	7
Mathews et al., 2015	★★★★	★	★★	7
Schild et al., 2019	★★★	★	★★★	7
Viswanath et al., 2018	★★★	★	★★	6
Koch et al., 2016	★★★★	★	★★	7
Viswanath et al., 2018	★★★	★	★★	6
Osawa et al., 2019	★★★★	★	★	6
Loskutov et al/. 2018	★★★	★	★★	6
Oancea-Castillo et al., 2017	★★★	★	★★	6
Bien-Moller et al., 2015	★★★★	★★	★★	8
Doan et al., 2017	★★★★	★	★★★	8
Riccitelli et al., 2013	★★★	★	★★	6
Bernhart et al., 2015	★★★★	★	★★	7
Li et al., 2015	★★★	★	★★	6
Dowdy et al., 2020	★★★	★	★★	6
Abuhusain et al., 2013	★★★	★★	★★	7
Abdel Hadi et al., 2018	★★★	★	★★	6
Bassi et al., 2021	★★★	★	★★	6
Doan et al., 2017	★★★	★	★★	6
Giussani et al., 2012	★★★	★	★★	6
Jung et al., 2016	★★★	★	★★	6
Wang et al., 2017	★★★	★	★★	6
Noack et al., 2014	★★★	★	★★	6
Romero-Ramirez et al., 2015	★★★	★	★★	6
Fleurence et al., 2016	★★★	★	★★	6
Yeh et al., 2016	★★★	★	★★★	7
Ermini et al., 2017	★★★★	★	★★★	8
Ohkawa et al., 2015	★★★★	★	★★★	8
Fabris et al., 2017	★★★★	★★	★★	8
Fabris et al., 2021	★★★★	★★	★★	8
Wingerter et al., 2021	★★★★	★	★★	7
Kundu et al., 2016	★★★	★	★★	6
Geng et al., 2016	★★★★	★	★★★	8
Eibinger et al., 2013	★★★★	★	★★	7
Cigliano et al., 2019	★★★★	★	★★	7
Li et al., 2020	★★★	★★	★	6
Clarion et al., 2012	★★★	★	★★	6
Emanuelsson et al., 2018	★★★	★	★★	6
McConnell et al., 2018	★★★★	★	★★	7
Cataldi et al., 2020	★★★★	★	★★	7
Samadi et al., 2017	★★★★	★	★★	7
Guo et al., 2013	★★★	★	★★	6
Cabodevilla et al., 2013	★★★★	★	★★	7
Abramczyk et al., 2021	★★★★	★	★★	7
Depciuch et al., 2019	★★★★	★★	★★	8
Lee et al., 2019	★★★★	★★	★★	8

Jothi et al., 2020	★★★★	★★	★★	8
Shao et al., 2014	★★★★	★	★★	7
Cuperlovic-Culf et al., 2012	★★★★	★	★★	7
Izquierdo-Garcia et al., 2015	★★★★	★	★★	7
Madhu et al., 2017	★★★★	★	★	6
Jalbert et al., 2017	★★★★	★	★★	7
Jarmusch et al., 2015	★★★★	★	★★	7
Kucharzewska et al., 2015	★★★★	★	★★	7
Haraszti et al., 2016	★★★★	★	★★	7
Antal et al., 2014	★★★★	★	★★	7
Li et al., 2018	★★★★	★★	★★	8
Jurchott et al., 2011	★★★	★	★★	6
St-Coeur et al., 2015	★★★★	★	★★	7
Pirro et al., 2017	★★★★	★	★★	7
Yu et al., 2020	★★★★	★★	★★	8
Zhang et al., 2017	★★★★	★	★★	7
Shakya et al., 2021	★★★★	★	★★★	8
Moren et al., 2016	★★★★	★	★★	7
Wood, 2018	★★★	★★	★★	7
Zigmont et al., 2015	★★★★	★★	★★	8
Liang et al., 2017	★★★★	★★	★★	8
Huang et al., 2017	★★★★	★★	★★	8
Agliano et al., 2017	★★★	★	★★★	7
Esmaeili et al., 2014	★★★★	★	★★★	8
Fack et al., 2017	★★★	★	★★★	7
Wenger et al., 2017	★★★★	★	★★★	8
Hattingen et al., 2013	★★★★	★★	★★	8
Seow et al., 2018	★★★★	★★	★	7
Pardieu et al., 2020	★★★★	★★	★	7
Ramli et al., 2015	★★★★	★★	★	7
Seow et al., 2019	★★★★	★★	★	7
Safronova et al., 2015	★★★★	★★	★	7
Imiela et al., 2020	★★★★	★	★★	7
Kopec et al., 2021	★★★★	★	★	6
Kast et al.,2015	★★★	★	★★	6
Kopec et al., 2019	★★★★	★	★	6
Salzillo et al., 2021	★★★★	★	★★★	8
Verma et al., 2018	★★★★	★★	★★★	9
Chakhoyan et al., 2017	★★★★	★★	★	7
Valentini et al., 2017	★★★★	★★	★	7
Bluml et al., 2016	★★★★	★★	★	7
Li et al., 2013	★★★★	★★	★	7
Craveiro et al., 2014	★★★★	★	★★★	8
Nelson et al., 2017	★★★★	★★	★	7
Toussaint et al., 2017	★★★★	★	★★★	8
Nakamura et al., 2018	★★★★	★	★	6
Park et al., 2014	★★★	★	★★★	7
Durmo et al., 2018	★★★★	★★	★	7
Palma et al., 2020	★★★★	★	★★	7
Delgado-Goni et al., 2016	★★★★	★	★★★	8
Hnilicova et al.,2017	★★★★	★★	★	7

Shang et al., 2011	★★★★	★	★	6
Ozturk-Isik et al., 2012	★★★★	★	★	6
Yao et al., 2021	★★★★	★	★	6
Hangel et al., 2020	★★★★	★★	★	7
Mohamed Fawzy et al., 2016	★★★★	★	★	6
Akagi et al., 2019	★★★★	★★	★	7
Luks et al., 2018	★★★★	★	★	6
Zeng et al., 2011	★★★★	★★	★	7
Kaddah & Khalil, 2014	★★★★	★★	★	7
Novak et al., 2014	★★★★	★★	★	7
Wilson et al., 2013	★★★★	★	★	6
Orphanidou-Vlachou et al., 2013	★★★★	★	★	6
Postma et al., 2011	★★★★	★	★	6
Porto et al., 2011	★★★★	★★	★	7
Bernabeu-Sanz et al., 2020	★★★★	★★	★	7
Aburano et al., 2015	★★★★	★★	★	7
Yamasaki et al., 2014	★★★★	★★	★	7
Mlynarik et al., 2011	★★★★	★	★★★	8
Wang et al., 2017	★★★★	★	★★★	8
Mora et al., 2018	★★★★	★★	★	7
Hulsey et al., 2014	★★★★	★	★★★	8
Sawlani et al., 2012	★★★★	★★	★	7
Vettukattil et al., 2012	★★★★	★★	★	7
Porto et al., 2012	★★★★	★★	★	7
Fujita et al., 2020	★★★★	★★	★	7
Goryawala et al., 2019	★★★★	★★	★	7
Wehrl et al., 2013	★★★★	★	★★★	8
Ramm et al, 2011	★★★★	★	★★	7
Li et al., 2018	★★★★	★★	★	7
Elkhaled et al., 2013	★★★★	★	★	6
Hattingen et al., 2011	★★★★	★★	★	7
Galijasevic et al., 2021	★★★★	★★	★	7
Mirbahai et al., 2012	★★★★	★	★★	7
Martin-Sitjar et al., 2012	★★★★	★	★★	7
Kampa et al., 2020	★★★★	★★	★	7
Gularyan et al., 2020	★★★★	★★	★	7
Calligaris et al., 2015	★★★★	★	★	6
Henderson et al., 2020	★★★★	★	★★★	8
Im et al., 2020	★★★★	★★	★★	8
Zhai et al., 2019	★★★★	★★	★	7
Anna et al., 2017	★★★	★	★	5
Abramczyk & Imiela., 2018	★★★★	★★	★	7
Moren et al., 2015	★★★★	★	★	6
Ballester et al., 2018	★★★	★★	★	6
Rogachev et al., 2021	★★★★	★	★	6
Eberlin et al., 2011	★★★★	★	★	6
Gilard et al., 2021	★★★★	★★	★	7

★ star was awarded for the study which meet the requirement for study criteria. The scores of NOS were ranged from 0 star (lowest score) to 9 stars (highest score). A study with a NOS score higher than 5 was recognised as a high-quality study [16].

Table S3. List of abbreviations for Figure 4.

Abbreviation	Systematic Name	Common Name
FA4:0	Butanoic acid	Butyric acid
FA8:0	Octanoic acid	Caprylic acid
FA10:0	Decanoic acid	Capric acid
FA12:0	Dodecanoic acid	Lauric acid
FA16:0	Hexadecanoic acid	Palmitic acid
FA18:0	Octadecanoic acid	Stearic acid
FA18:1	9Z-octadecenoic acid	Oleic acid
FA18:3	9Z,12Z,15Z-octadecatrienoic acid	Alpha-Linolenic acid
FA20:5	5Z,8Z,11Z,14Z,17Z-eicopentaenoic acid	Eicosapentaenoic acid
FA22:6	4Z,7Z,10Z,13Z,16Z,19Z-docosahexaenoic acid	Docosahexaenoic acid
CAR18:0	O-octadecanoyl-R-carnitine	Stearoylcarnitine
(FA20:4;O3) PGD2	9S,15S-dihydro-11-oxo-5Z,13E-prostadienoic acid	Prostaglandin D2
(FA20:4;O3) PGE2	9-oxo-11R,15S-dihydroxy-5Z,13E-prostadienoic acid	Prostaglandin E2
MG20:4	2-(5Z,8Z,11Z,14Z-eicosatetraenoyl)-sn-glycerol	2-Arachidonylglycerol
DG	Diacyl-sn-glycerol	Diacylglycerols
TG	Triacyl-sn-glycerol	Triacylglycerols
PA	1,2-diacyl-sn-glycero-3-phosphate	Phosphatidic acid
PC	1,2-diacyl-sn-glycero-3-phosphocholine	Phosphatidylcholine
PE	1,2-diacyl-sn-glycero-3-phosphoethanolamine	Phosphatidylethanolamine
PI	1,2-diacyl-sn-glycero-3-phospho-(1′-myo-inositol)	Phosphatidylinositol
PG	1,2-diacyl-sn-glycero-3-phospho-(1′-sn-glycerol)	Phosphatidylglycerol
LPA	1-acyl-sn-lycero-3-phosphate	Lysophosphatidic acid
LPE	1-acyl-sn-lycero-3-phosphoethanolamine	Lysophosphatidylethanolamine
LPC	1-acyl-sn-lycero-3-phosphocholine	Lysophophatidylcholine
S1P	Sphing-4-enine-1-phosphate	Sphingosine-1-phosphate
Cer (Cer20:1;O2)	N-acyl-sphing-4-enine	Ceramide
C2 Cer	N-(acetyl)-sphing-4-enine	C2-Ceramide
(Cer36:1;O2) C18 Cer	N-(octadecanoyl)-sphing-4-enine	C18 Ceramide
dhCer	N-acyl-sphinganine	Dihydroceramide
(SPB18:0;O2) dhSph	Sphinganine	Dihydrosphingosine
SM	N-acyl-sphing-4-enine-1-phosphocholine	Sphingomyelin
(NDMS	N,N-dimethylsphing-4-enine	N,N-dimethylsphingosine (NDMS)
Cerd(d42:1) C24 Cer	N-(tetracosanoyl)-ceramide	C24 Cer
OAcGD2		
GD2	GalNAcbeta1-4(NeuAcalpha2-8NeuAcalpha2-3)Galbeta1-4Glcbeta-Cer	Ganglioside GD2
GD3	NeuAcalpha2-8NeuAcalpha2-3Galbeta1-4Glcbeta-Cer	Ganglioside GD2
GM2	GalNAcbeta1-4(NeuAcalpha2-3)Galbeta1-4Glcbeta-Cer	Ganglioside GM2
CE	cholest-5-en-3β-yl dodecanoate	Cholesterol ester
ST21:1;O4	3α,11β,21-5α-trihydroxy-pregnane-20-one	Tetrahydrocorticosterone
CH25H	cholest-5-en-3β-ol	Cholesterol
(ST 27:1;O2) 24S-OHC	cholest-5-en-3β,24S-diol	24S-hydroxy-cholesterol
Vit D3	(5Z,7E)-(3S)-9,10-seco-5,7,10(19)-cholestatrien-3-ol	Vitamin D3

Table S4. Chemical structures of the studied lipids. The chemical structures and lipid details were acquired from LIPID MAPS, Kyoto Encyclopedia of Genes and Genomes (KEGG), and PubChem databases.

Common Name	Systematic Name	Chemical Formula	Chemical Structures	Lipid Roles
**Fatty Acyl (FA)**				
Butyric acid	Butanoic acid	$C_4H_8O_2$	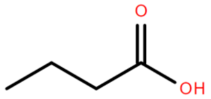	Carcinogenic lipid
Caprylic acid	Octanoic acid	$C_8H_{16}O_2$	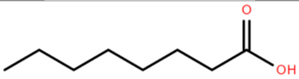	Carcinogenic lipid
Capric acid	Decanoic acid	$C_{10}H_{20}O_2$	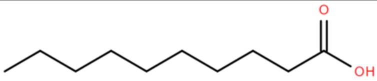	Carcinogenic lipid
Lauric acid	Dodecanoic acid	$C_{12}H_{24}O_2$	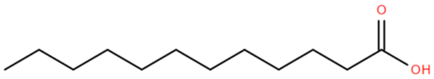	Carcinogenic lipid
Palmitic acid	Hexadecanoic acid	$C_{16}H_{32}O_2$	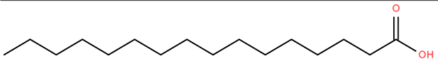	Carcinogenic & Anti-carcinogenic lipid
Stearic acid	Octadecanoic acid	$C_{18}H_{36}O_2$	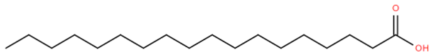	Carcinogenic & Anti-carcinogenic lipid
Oleic acid	9Z-octadecenoic acid	$C_{18}H_{34}O_2$	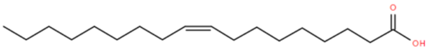	Carcinogenic lipid
Gamma-Linolenic acid	6Z,9Z,12Z-octadecatrienoic acid	$C_{18}H_{30}O_2$	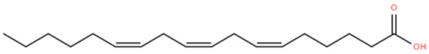	Carcinogenic lipid & Anti-carcinogenic lipid
Eicosapentaenoic acid	5Z,8Z,11Z,14Z,17Z-eicosapentaenoic acid	$C_{20}H_{30}O_2$	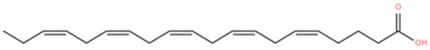	Anti-carcinogenic lipid
Arachidonic acid	5Z,8Z,11Z,14Z-eicosatetraenoic acid	$C_{20}H_{32}O_2$	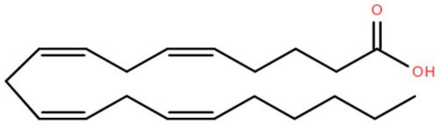	Carcinogenic lipid
Docosahexaenoic acid	4Z,7Z,10Z,13Z,16Z,19Z-docosahexaenoic acid	$C_{22}H_{32}O_2$	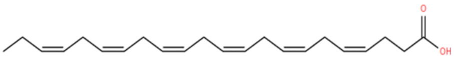	Anti-carcinogenic lipid
Lipoic acid	1,2-dithiolane-3R-pentanoic acid	$C_8H_{14}O_2S_2$	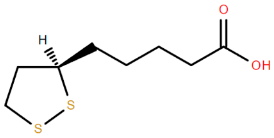	Anti-carcinogenic lipid
Stearoylcarnitine	O-octadecanoyl-R-carnitine	$C_{25}H_{49}NO_4$	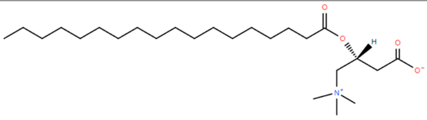	Carcinogenic lipid
Prostaglandin D2	9S,15S-dihydroxy-11-oxo-5Z,13E-prostadienoic acid	$C_{20}H_{32}O_5$	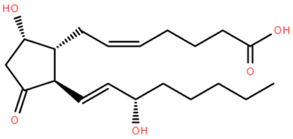	Carcinogenic lipid
Prostaglandin E2	9-oxo-11R,15S-dihydroxy-5Z,13E-prostadienoic acid	$C_{20}H_{32}O_5$	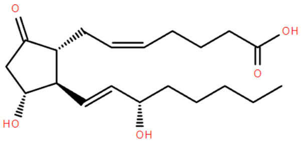	Carcinogenic lipid
**Glycerolipid (GL)**				
2-Arachidonoylglycerol	2-(5Z,8Z,11Z,14Z-eicosatetraenoyl)-sn-glycerol	$C_{23}H_{38}O_4$	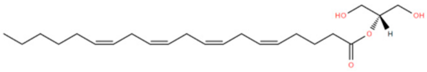	Carcinogenic lipid
Diacylglycerol	1-(hexadecanoyloxy)-3-hydroxypropan-2-yl-	$C_{37}H_{70}O_5$	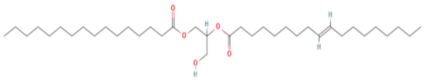	Carcinogenic lipid

	octadec-9-enoate			
**Glycerophospholipid (GP)**				
Phosphatidic acid	1,2-diacyl-sn-glycero-3-phosphate	$C_5H_7O_8PR2$	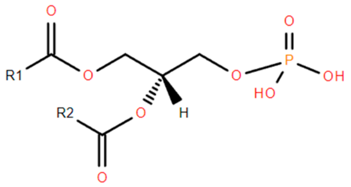	Carcinogenic lipid
Phosphatidylcholine	1,2-diacyl-sn-glycero-3-phosphocholine	$C_{10}H_{18}NO_8PR2$	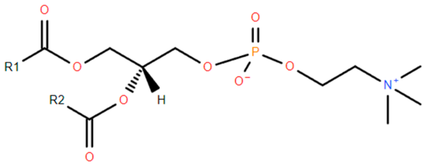	Carcinogenic lipid
Phosphatidylethanolamine	1,2-diacyl-sn-glycero-3-phosphoethanolamine	$C_7H_{12}NO_8PR2$	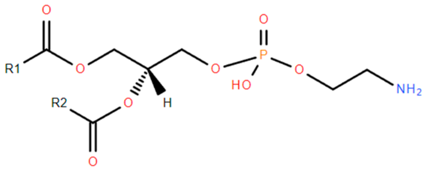	Carcinogenic lipid
Phosphatidylinositol	1,2-diacyl-sn-glycero-3-phospho-(1'-myo-inositol)	$C_{11}H_{17}O_{13}PR2$	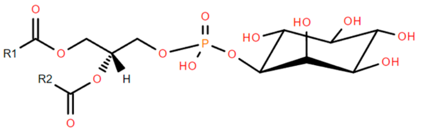	Carcinogenic lipid
Phosphatidylglycerol	1,2-diacyl-sn-glycero-3-phospho-(1'-sn-glycerol)	$C_8H_{13}O_{10}PR2$	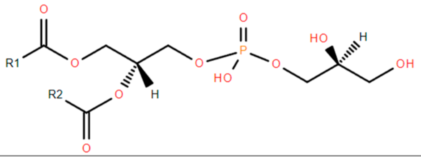	Carcinogenic lipid
Lysophosphatidic acid	1-acyl-sn-glycero-3-phosphate	$C_4H_8O_7PR$	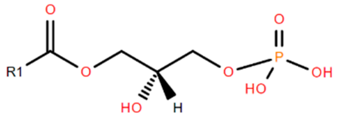	Carcinogenic lipid
Lysophosphatidylcholine	1-acyl-sn-glycero-3-phosphocholine	$C_9H_{20}NO_7PR$	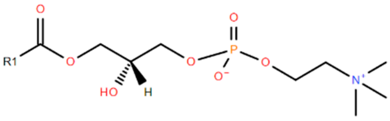	Carcinogenic lipid
Lysophosphatidylethanolamine	1-acyl-sn-glycero-3-phosphoethanolamine	$C_6H_{13}NO_7PR$	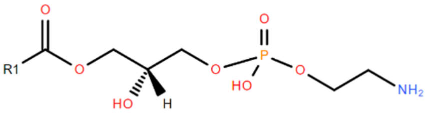	Carcinogenic lipid
**Sphingolipid (SP)**				
Sphingosine-1-phosphate	Sphing-4-enine-1-phosphate	$C_{18}H_{38}NO_5P$	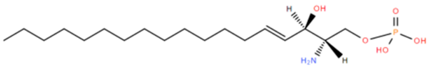	Carcinogenic lipid
Ceramide	N-acyl-sphing-4-enine	$C_{19}H_{36}NO_3R$	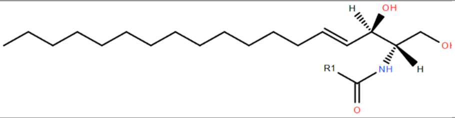	Carcinogenic lipid
C2 Ceramide	N-(acetyl)-sphing-4-enine	$C_{20}H_{39}NO_3$	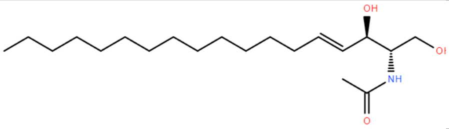	Anti-carcinogenic lipid
C18 Ceramide	N-stearoyl-D-erythro-sphingosine	$C_{36}H_{71}NO_3$	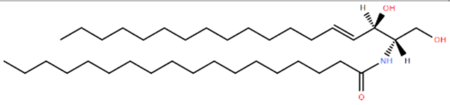	Anti-carcinogenic lipid

Dihydroceramide	N-acyl-sphinganine	$C_{19}H_{38}NO_3R$	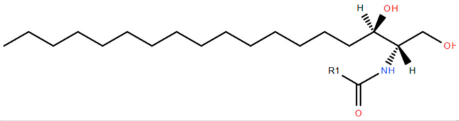	Anti-carcinogenic lipid
Dihydrosphingosine	Sphinganine	$C_{18}H_{39}NO_2$	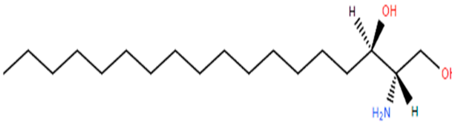	Carcinogenic lipid & Anti-carcinogenic lipid
Sphingomyelin	N-acyl-sphing-4-enine-1-phosphocholine	$C_{24}H_{49}N_2O_6PR$	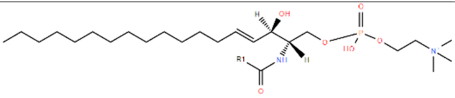	Carcinogenic lipid
N,N-dimethylsphingosine	N,N-dimethylsphing-4-enine	$C_{20}H_{41}NO_2$	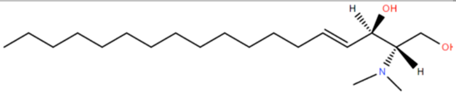	Carcinogenic lipid
Sphingosine	Sphing-4-enine	$C_{18}H_{37}NO_2$	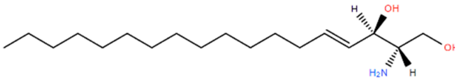	Carcinogenic lipid
Disialoganglioside GD2	GalNAcβ1-4(NeuAcα2-8NeuAcα2-3)Galβ1-4Glcβ-Cer	$C_{74}H_{134}N_4O_{32}$	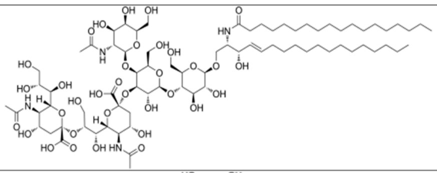	Carcinogenic lipid
Ganglioside GM2	GalNAcβ1-4(NeuAcα2-3)Galβ1-4Glcβ-Cer	$C_{68}H_{123}N_3O_{26}$	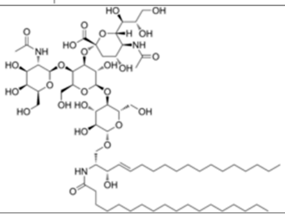	Carcinogenic lipid
**Sterol Lipid (ST)**				
Cholesterol Ester	cholest-5-en-3β-yl nonadecanoate	$C_{28}H_{45}O_2R$	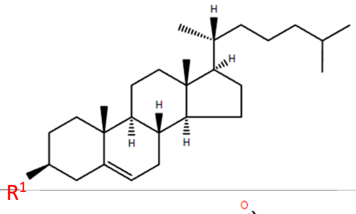	Carcinogenic lipid
Tetrahydrocorticosterone	3α,11β,21-5α-trihydroxy-pregnane-20-one	$C_{21}H_{34}O_4$	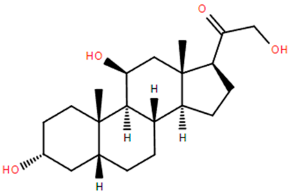	Carcinogenic lipid
Cholesterol	cholest-5-en-3β-ol	$C_{27}H_{46}O$	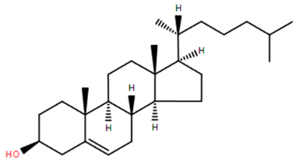	Carcinogenic lipid
24S-hydroxy-cholesterol	cholest-5-en-3β,24S-diol	$C_{27}H_{46}O_2$	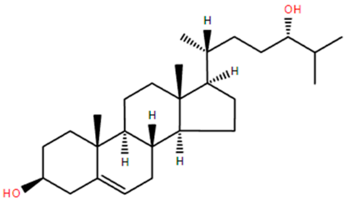	Carcinogenic lipid

7β-Hydroxycholesterol	5-cholestene-3β,7β-diol	$C_{27}H_{46}O_2$	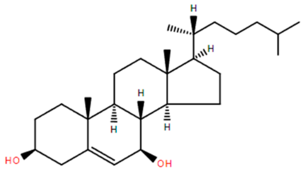	Anti-carcinogenic lipid
Vitamin D3	(5Z,7E)-(3S)-9,10-seco-5,7,10(19)-cholestatrien-3-ol	$C_{27}H_{44}O$	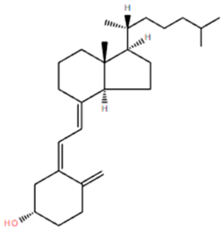	Anti-carcinogenic lipid
**Prenol Lipid (PR)**				
Oleanoic acid	28-hydroxy-28-oxoolean-12-en-3β-yl β-D-glucopyranosiduronic acid	$C_{36}H_{56}O_9$	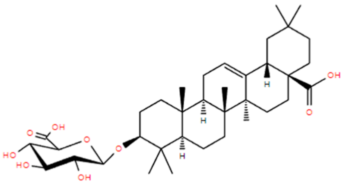	Anti-carcinogenic lipid

*Abbreviation: C carbon; H hydrogen; O oxygen; N nitrogen; P phosphate; S sulphur; OH hydroxyl group; NH_2 amino group; R^1 First subgroup; R^2 second subgroup; R^3 third subgroup
